# A mechanism-based pathway toward administering highly active N-phage cocktails

**DOI:** 10.3389/fmicb.2023.1292618

**Published:** 2023-11-15

**Authors:** Nicholas M. Smith, Thomas D. Nguyen, Wai Hoe Chin, Jacob T. Sanborn, Harriet de Souza, Brian M. Ho, Tiffany Luong, Dwayne R. Roach

**Affiliations:** ^1^Division of Clinical and Translational Therapeutics, School of Pharmacy and Pharmaceutical Sciences, University at Buffalo, Buffalo, New York, NY, United States; ^2^Department of Biology, San Diego State University, San Diego, CA, United States

**Keywords:** *Pseudomonas aeruginosa*, phage therapy, hollow fiber infection model, pharmacokinetics, pharmacodynamics, mathematical modeling, phage cocktails, treatment optimization

## Abstract

Bacteriophage (phage) therapy is being explored as a possible response to the antimicrobial resistance public health emergency. Administering a mixture of different phage types as a cocktail is one proposed strategy for therapeutic applications, but the optimal method for formulating phage cocktails remains a major challenge. Each phage strain has complex pharmacokinetic/pharmacodynamic (PK/PD) properties which depend on the nano-scale size, target-mediated, self-dosing nature of each phage strain, and rapid selection of resistant subpopulations. The objective of this study was to explore the pharmacodynamics (PD) of three unique and clinically relevant anti-*Pseudomonas* phages after simulation of dynamic dosing strategies. The Hollow Fiber Infection Model (HFIM) is an *in vitro* system that mimics *in vivo* pharmacokinetics (PK) with high fidelity, providing an opportunity to quantify phage and bacteria concentration profiles over clinical time scales with rich sampling. Exogenous monotherapy-bolus (producing *max* concentrations of *C*_max_ = 7 log_10_ PFU/mL) regimens of phages LUZ19, PYO2, and E215 produced *Pseudomonas aeruginosa* nadirs of 0, 2.14, or 2.99 log_10_ CFU/mL after 6 h of treatment, respectively. Exogenous combination therapy bolus regimens (LUZ19 + PYO2 or LUZ19 + E215) resulted in bacterial reduction to <2 log_10_ CFU/mL. In contrast, monotherapy as a continuous infusion (producing a *steady-state* concentration of *C*_ss,avg_ = 2 log_10_PFU/mL) was less effective at reducing bacterial densities. Specifically, PYO2 failed to reduce bacterial density. Next, a mechanism-based mathematical model was developed to describe phage pharmacodynamics, phage–phage competition, and phage-dependent adaptive phage resistance. Monte Carlo simulations supported bolus dose regimens, predicting lower bacterial counts with bolus dosing as compared to prolonged phage infusions. Together, *in vitro* and *in silico* evaluation of the time course of phage pharmacodynamics will better guide optimal patterns of administration of individual phages as a cocktail.

## Introduction

Phage therapy is the treatment of infectious disease using infusions of bacterial viruses called bacteriophages (phages). The origins of phage therapy can be traced back to the early 1920s with the work of d’Herelle (1917), Summers (2012). However, the development of phages as antibacterial agents waned as highly active small molecule antibiotics became commonplace (Stennett et al., 2022). There are many barriers to the success of phage therapy including drug delivery strategies, manufacturing, clinical dosing strategies, and rapid development of resistance (Oechslin, 2018; Suh et al., 2022; Champagne-Jorgensen et al., 2023; Petrovic Fabijan et al., 2023). Regarding the clinical pharmacology of phages, dosing strategies are unclear given the complexities of phage pharmacodynamics *in vivo* and the effects of phage resistance on efficacy and longevity of any developed phage therapeutic (Abedon, 2019; Hatfull et al., 2022; Nang et al., 2023). With the emergence of extensively and pan drug resistant bacteria, phage therapies are being revitalized as one of the few alternative strategies to combat bacterial diseases (Magiorakos et al., 2012; Gottig et al., 2014; Chen et al., 2017; Tacconelli et al., 2018; CDC, 2019).

To maximize bacterial killing and minimize proliferation of resistant subpopulations, often two or more phage strains with different viral properties are used in combination as a cocktail, here referred to as an “N-phage cocktail.” An optimal N-phage cocktail can both increase spectrum of activity and potentially prevent the selection of resistant mutants by, for example, optimizing selection of unique bacterial cell surface receptors (Abedon et al., 2021; Li et al., 2022; Naknaen et al., 2023). Phages however have multiple infective properties that may also affect the time-course of their pharmacology, for example adsorption rate, burst size, latent period, genome content/size (surrogate for viral complexity), and mutation frequency (Kannoly et al., 2022; Abedon, 2023). These properties vary by phage type and target host bacteria (Abedon et al., 2003; Forti et al., 2018; Nabergoj et al., 2018). As a result, phages represent a class of anti-bacterials where the traditional pharmacokinetic/pharmacodynamic (PK/PD) indices of action are no longer applicable (e.g., percent time above MIC, area under the concentration-time curve, or maximum concentration). Thus, in the evaluation of conventional antibiotics, drug exposure is independent from the exposure-response relationship. Whereas the self-replication of phages complicates the characterization of phage-specific pharmacodynamics. With phage therapy, exposure is no longer an independent variable and lysis outcomes of phage-bacteria interactions are directly correlated to gross anti-bacterial activity (Venturini et al., 2022; Nang et al., 2023). Therefore, developing an efficient N-phage cocktail requires optimal selection and administration of multiple phage strains, leveraging their individual viral properties. In addition, strain–strain interactions can also influence cocktail performance.

The complexity of phage dosing is largely related to their nano-scale sizes, which influences drug disposition. Target-mediated drug disposition (TMDD) is defined as the ability of a drug binding to its receptor to alter its own disposition, elimination, or a combination of both (Levy, 1994). The concept was further refined with the emergence and proliferation of biologics, such as monoclonal antibodies (Dua et al., 2015; An, 2020). However, the unmodified TMDD framework does not accurately characterize the unique aspects of phage therapy. Indeed, TMDD already accounts for an initial rapid, target-dependent decrease in phage concentration due to viral adsorption. However, phage lytic replication (i.e., auto-dosing) is also target-dependent, which results in an increase in phage concentration over time. Therefore, employing a target-mediated phage disposition (TMPD) framework captures the self-replicating mode of phage action and can account for phages’ nonlinear PK.

Moreover, quantifying phage parameters of infectivity can also be accomplished using a mechanism-based mathematical model of phage PD using *in vitro* phage activity patterns. For instance, PK/PD optimization of an N-phage cocktail should consider both the properties of each phage strain and measured phage strain–strain interaction. Previous models of individual phage activities have largely implemented a susceptible, infected, and recovered subpopulations of bacteria (SIR) model structure (Cairns et al., 2009; Landersdorfer et al., 2013; Styles et al., 2021). This foundation model can be adapted with modern concepts of drug–drug interactions to facilitate description of individual phage activity alongside phage–phage interaction (e.g., additive, synergistic, or antagonistic).

Preclinical PK/PD models play a critical role in designing human dosage regimens and are essential tools for drug development. For antibacterial PK/PD, the *in vitro* hollow fiber infection model (HFIM) can provide valuable and complementary information for dose selection and translation of antimicrobials from the laboratory to humans (Lodise et al., 2020, 2022). The HFIM is an ideally suited *in vitro* model for evaluating phage PK/PD and identify target concentrations that best predict bacterial killing and resistance prevention. That is, the HFIM can be leveraged to develop recommendations, identify common pitfalls, and describe the applications, strengths, and limitations of translational approaches over clinically relevant timeframes (Lodise et al., 2020, 2022). This system can simulate virtually any time course of phage concentrations for one or multiple phages with the same or different half-lives. The hollow fiber cartridge has a large surface-to-volume ratio providing optimized growth conditions for bacteria and waste products are continually removed. Ultimately, combining HFIM data of individual phage activity and phage–phage pairs can be leveraged with mechanism-based mathematical modeling to devise optimal phage cocktails both in terms of constituent phage types and phage type administration pattern.

Because the widespread implementation of phage therapy in routine clinical practice is impeded by the scarcity of clinical data, such as randomized controlled trials (RCTs), characterizing preclinical PK/PD relationships of phages in needed to adapt future research and therapy development. In this study, we used a mechanism-based PK/PD evaluation of three clinically relevant virulent phages (LUZ19, PYO2, and E215) that all infect the opportunistic pathogen *Pseudomonas aeruginosa*. This bacterium can cause several types of human infections and is often resistant to many classes of antibiotics and therapeutic agents, because of its problematic nature during infection it has become a common treatment target for phage therapy (Lin et al., 2017; Luong et al., 2020; Uyttebroek et al., 2022). Here, we utilized a preclinical PK/PD infection model to elucidate N-phage cocktail exposure-response relationships and to subsequently identify optimal administration methods.

## Material and methods

### Bacteria, phage, and media

*Pseudomonas aeruginosa* laboratory strain PAO1 was used for all experiments. All culturing was conducted in Mueller–Hinton Broth (Mg^2+^ 25 mg/L, Ca^2+^ 12.5 mg/L) or agar (Becton Dicksinson, Sparks, MD) at 37°C. Agar (1.5%) was added to MHB for solid growth medium. Previously characterized virulent podovirus LUZ19 (Lavigne et al., 2013), podovirus PYO2 (Forti et al., 2018), virulent myovirus E215 (Forti et al., 2018) were freshly cultured and purified before treatment, using a methodology previously described (Luong et al., 2020). Briefly, phage lysates were sterilized by 2× high-speed centrifugation and 0.2 μm dead-end filtration. Fresh phage stocks were prepared every 48 h and stored, protected from light, at 4°C.

### Hollow fiber infection model

PAO1 was studied over 7 d using polyvinylidene fibers (C2025, FiberCell, New Market, MD) with 0.1 μm pore size. The system half-life was fixed to 2 h based on assessment of limited available literature concerning *in vivo* PK (Bichet et al., 2021; Suh et al., 2022; Nang et al., 2023). The total system volume of distribution was 125 mL with a clearance rate of 0.7 mL/min. For bolus strategies with a target *C*_max_ = 7 log_10_ (PFU/mL), a stock concentration of 11.6 log_10_ PFU/mL was used. For continuous infusion strategies with a target *C*_ss,avg_ = 2 log_10_ PFU/mL, a stock concentration of 6.58 log_10_ (PFU/mL) was used.

Serial samples were collected for enumeration of bacteria and phages during treatment. Bacteria were quantified from samples obtained from the extracapillary space of the HFIM cartridge. Bacteria were quantified by serially diluting samples and plating 50 μL on MHA then incubating for 24 h prior to automated enumeration (Protos, Synbiosis). By comparison, phages were quantified in both the central reservoir and the extracapillary space of the HFIM cartridge to fully account for full disposition of phage in the system. Individual phage strains were distinguished through quantification on PAO1-resistant mutant lawns (i.e., PAO1_LUZ19R_, PAO1_E215R_, and PAO1_PYO2R_). Phages were quantified by double aliquot 48-spot serial titration, as previously described. Briefly, phage-resistant PAO1 mutant cultured at OD_600_ 0.2 was lawned and dried over agar medium before spotting two identical 8-well columns of tenfold serial diluted phage 4 μL samples. Agar plates were incubated overnight at 37°C before plaque enumeration. Data were graphed using R (version 4.3.0) with no observable counts (i.e., 0 PFU) plotted as 0 log_10_ PFU/mL for visualization purposes.

### *In vitro* pharmacokinetics and treatment regimens

Regimens were designed to discriminate between phages administered and those produced endogenously during treatment. This controlled for the dynamic administration of doses over time to the HFIM, as well as support parameter identifiability in the mathematical modeling. Choice of regimen was also made to ensure that eventual mathematical modeling would be able to identify PK parameters related to disposition to the cartridge along with PD parameters. To accomplish these goals, single bolus dosing and continuous infusion strategies were implemented. Single bolus dosing provides a clear peak concentration (*C*_max_), followed by exponentially declining counts following the system’s 2 h half-life. The dose utilized for bolus dosing was designed to empirically achieve a peak concentration of 7 log_10_ (PFU/mL), which corresponds to a multiplicity of infection of 1:10. By comparison, keeping the phage concentration constant with continuous infusion can ensure that the phage concentration more readily reaches a steady state. Dosing was selected to achieve a steady-state concentration of 10^2^ PFU/mL to maximize the dynamic range that we can quantify endogenous production of phage. Using this method, concentrations observed >10^2^ PFU/mL were attributed to endogenous production. The 11 regimens are outlined in Table 1. Combination regimens were tested in duplicate and reported as the mean value.

**Table 1:** Regimens studied in the hollow fiber infection model.

	PYO2	E215	LUZ19
1	–	–	–
2	Continuous infusion Q48H (*C*_max_ = 10^7^ PFU/mL)		
3		Continuous infusion Q48H (*C*_ss,avg_ = 10^2^ PFU/mL)	
4			Continuous infusion Q48H (*C*_ss,avg_ = 10^2^ PFU/mL)
5	Bolus x1 (*C*_max_ = 10^7^ PFU/mL)		
6		Bolus x1 (*C*_max_ = 10^7^ PFU/mL)	
7			Bolus x1 (*C*_max_ = 10^7^ PFU/mL)
8		Continuous infusion Q48H (*C*_ss,avg_ = 10^2^ PFU/mL)	Continuous infusion Q48H (*C*_ss,avg_ = 10^2^ PFU/mL)
9	Continuous infusion Q48H (*C*_ss,avg_ = 10^2^ PFU/mL)		Continuous infusion Q48H (*C*_ss,avg_ = 10^2^ PFU/mL)
10		Bolus x1 (*C*_max_ = 10^7^ PFU/mL)	Bolus x1 (*C*_max_ = 10^7^ PFU/mL)
11	Bolus x1 (*C*_max_ = 10^7^ PFU/mL)		Bolus x1 (*C*_max_ = 10^7^ PFU/mL)

Bolus regimens were designed to produce a *C*_max_ of 10^7^PFU/mL, which would correspond to a predicted MOI of 1:10. By comparison, continuous infusion regimens were designed to produce the eventual steady-state concentration of 10^2^ PFU/mL, to enhance the dynamic range of phage detection to better discriminate between endogenous production and exogenous administration.

### Mechanism-based mathematical modeling

A mechanism-based model was developed with ordinary differential equations (ODE) to quantify the PD of phage monotherapy and 2-phage cocktails using Monolix version 2023R1 (Lixoft, Antony, FR). Model estimates were determined via the stochastic approximation expectation maximization algorithm with standard errors calculated through linearization of the Fisher Information Matrix. Inter-experimental variability was handled by empirically fixing random effects for parameters dependent on experimental set-up or growth conditions to 5% CV (LGCFUMX, LGINOC, MGT, *τ*, *N*). For all other parameters, the random effects were fixed to 0.

Given the pharmacodynamic complexity of the phage infection and replication, we next developed a mechanism-based Susceptible-Infected-Recovered (SIR) mathematical model. Importantly, we updated the SIR model to include adaptive phage resistance, which we believe is enviable to occur during phage therapy (Cairns et al., 2009; Jacobs et al., 2016). To produce the most parsimonious model, resistance to PYO2 or E215 was modeled as a single process given both phages target the O-antigen region of the LPS, which was dependent on the concentration of phage-infected host cells, namely virocells. Therefore, only a single resistant sub-population was used to describe LUZ19-susceptible, PYO2/E215-resistant cells.

We describe PAO1 as a single initial subpopulation of cells susceptible to all three phage strains and the cells growth rate was modeled as mean generation time (MGT) (Smith et al., 2022). In addition, a logistic growth model with a shape parameter was used to predict the bacterial population’s saturable growth to its carrying capacity. As mentioned, phage resistance was modeled as an adaptive process, where exposure to a given phage strain selects for resistant mutants and a unique growth rate was estimated MGT of a triple-phage resistant subpopulation to account for mutation fitness loss.

Phage action characterized by adsorption and bacterial conversion to virocells were both described by a second order rate constant. Virocells were modeled as maturing through a series of transit compartments by a first order rate constant, *k*_tr_, which, by Eqs. (1)–(3), was fit in terms of the mean transit time through all compartments (Supplemental Material). The initial estimates of mean transit time were set to the previously measured one-step latent periods for each phage strain (Lavigne et al., 2013; Forti et al., 2018).

Phage–phage interactions were characterized as collateral effects of virocells presence of one phage on the adsorption rate of the other phage. This was accomplished by utilizing the virocell concentration in the fourth transit compartment to drive a Hill-type function. The EC_50_ is thus described as the concentration of virocells for 50% of maximum interaction. The shape parameter was fixed to 5 as it was found to greatly increase the model stability and produced an ‘on/off’-effect related to adaptive resistance and phage–phage interactions. Initially, all possible interactions were tested (i.e., PYO2 on LUZ19, E215 on LUZ19, LUZ19 on PYO2, or LUZ19 on E215). Interaction parameters were backward eliminated based on their respective Wald statistic.

Models were compared based changes in the objective function (−2•Log-likelihood) and diagnostic plots. Data below the limit of quantification (10^2^ CFU/mL or 10^2^ PFU/mL) were modeled as censored data, per the Monolix documentation. Data visualization was performed using ggplot2 and R version 4.2.2 (Fidler et al., 2018). Parameters were backwards eliminated, based on parameters that had the largest Wald-statistic.

### Monte Carlo simulation of different regimen structures

The final model was then leveraged to perform simulation-based studies testing different clinical administration strategies to identify the influence of dose and dose fractionation on the pharmacodynamic response. To test the ability to extend small molecule antibiotic dosing practices to the dosing of phages, Monte Carlo simulations were performed utilizing the mechanism-based model. First, dose escalation strategies were explored by simulating monotherapy with each phage with a dose of 3, 4, 5, 6, 7, 8, or 9 log_10_ (PFU)/day given as a single daily bolus over 48 h. Second, we simulated the effects of dose fractionation by utilizing a constant 7 log_10_ (PFU)/day dose divided across 1, 2, 3, 4, or 6 doses per day. Simulations were performed using RxODE in R (Fidler et al., 2018).

## Results

### Phage therapy in the hollow fiber infection model

**Figure 1:**
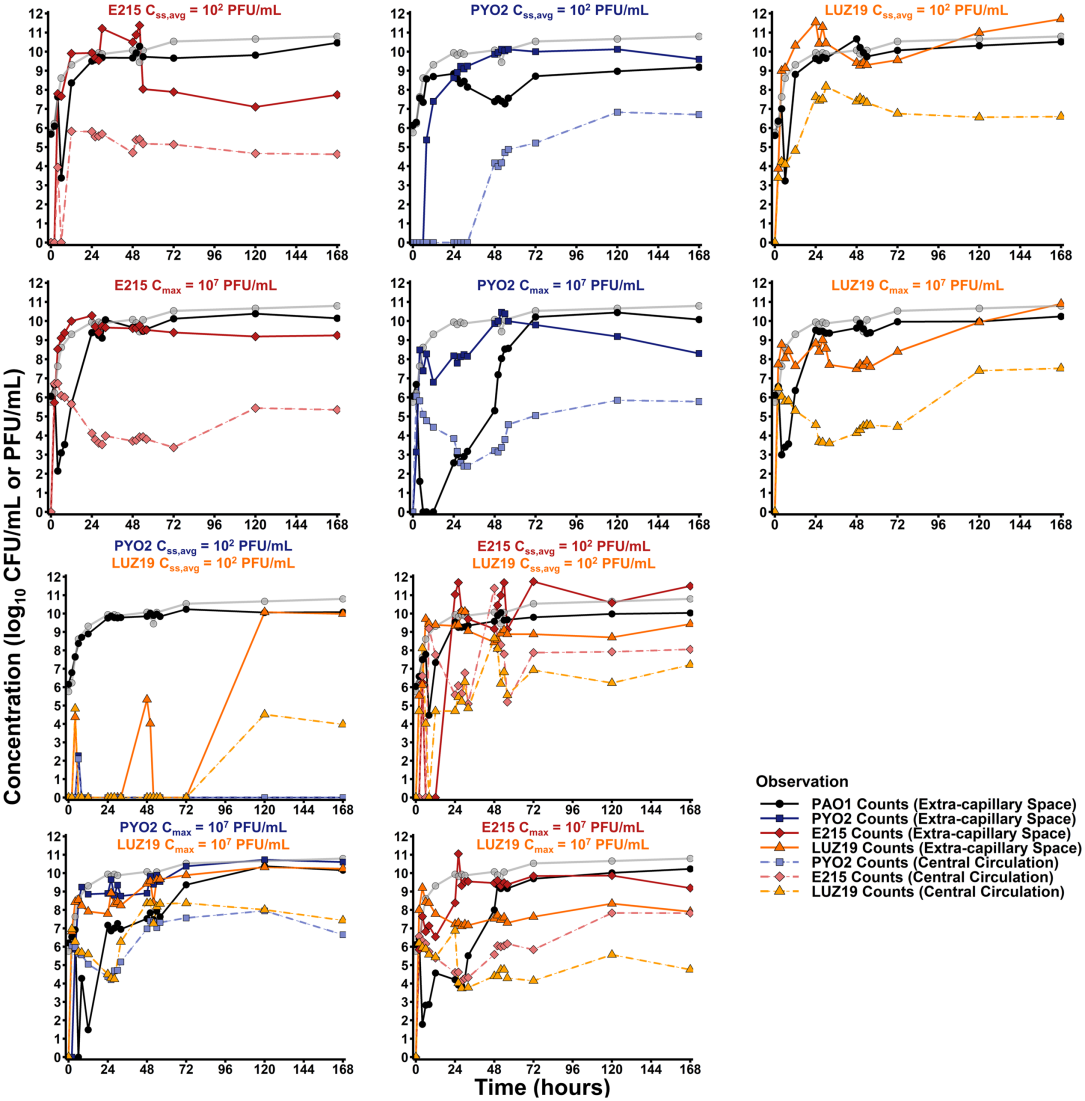
Hollow fiber infection model results. Total observed counts for PAO1 (black) for each regimen tested are overlayed with the growth control (gray). Total counts in the extra-capillary space/central circulation for E215 (dark red/light red), PYO2 (dark blue/light blue), or LUZ19 (dark orange/light orange) are reported with relevant experiments. Bolus regimens display clear first order elimination, as expected, from 0 to 24, when counts either plateau or increase due to endogenous production of phage.

To verify that the experimental set-up produced the targeted pharmacokinetics, phage concentrations in the central reservoir were analyzed by linear regression and found to decline with a 3.25, 2.54, and 3.08 h half-life for PYO2, E215, and LUZ19 (Figure 1; Supplementary Figure S1). However, the endogenous production of phage resulted in persistently elevated phage concentrations throughout 168 h. In contrast, phage concentrations from the continuous infusion regimens exhibited increased stochasticity for the first 4 h given the quantification limit of 2 log_10_ (CFU/mL), until endogenously produced phage could distribute from the cartridge back to the central reservoir.

The monotherapy control arms for all three phages were given as either bolus dosing or continuous infusion. Bolus dosing emulated a therapeutic strategy where selected phage concentrations would rapidly achieve a target MOI of 10. Using backward extrapolation from the 2 h sample, PYO2, E215, and LUZ19 were estimated as having peak concentrations of 6.64, 6.88, and 6.67 log_10_ (PFU/mL) in the central reservoir after bolus administration at 0 h, resulting in a measured MOI of 19. Monotherapy bolus regimens with PYO2, E215, or LUZ19 resulted in significant bactericidal activity as defined by >99.9% or 3 log_10_ (CFU/mL) reduction and resulted in minimum counts of 0, 2.14, or 2.99 log_10_ (CFU/mL), respectively. By comparison, continuous infusion regimens were designed to reach a steady-state concentration of 2 log_10_ (PFU/mL), which would take 10 h given the 2 h half-life of the system. In doing so, antibacterial effects from the continuous infusion regimens could be largely attributed to endogenous production of phages. The continuous infusion strategy resulted in less bacterial killing, for all three phage strains. That is, PYO2 monotherapy failed to produce any bacterial killing as continuous infusion, whereas E215 or LUZ19 monotherapy producing nadirs of 3.38 or 3.23 log_10_ (CFU/mL), respectively. Across all bactericidal regimens, peak bacterial killing was observed at 6 h, except for monotherapy bolus with LUZ19 which achieved peak killing effect at 4 h.

To test for phage–phage interactions, combination therapy was explored with either LPS-binding phage (i.e., PYO2 or E215) with the pili-binding phage (LUZ19). Duplicate runs of both combination regimens (i.e., PYO2 + LUZ19 and E215 + LUZ19) resulted in bactericidal activity (≥3 log_10_ CFU/mL reduction) an increased duration of bacterial suppression when utilizing bolus dosing, but both combination regimens exhibited nominal activity under continuous infusion. Single bolus dosing of PYO2 + LUZ19 achieved undetectable counts by 6 h then grew to the system’s carrying capacity after 24 h. By comparison, single bolus dosing of E215 + LUZ19 reached a nadir of 4.47 CFU/mL by 8 h, indicating potential antagonism in the case of simultaneous administration.

### Mechanism-based susceptible-infected-adaptively resistant mathematical model

**Figure 2:**
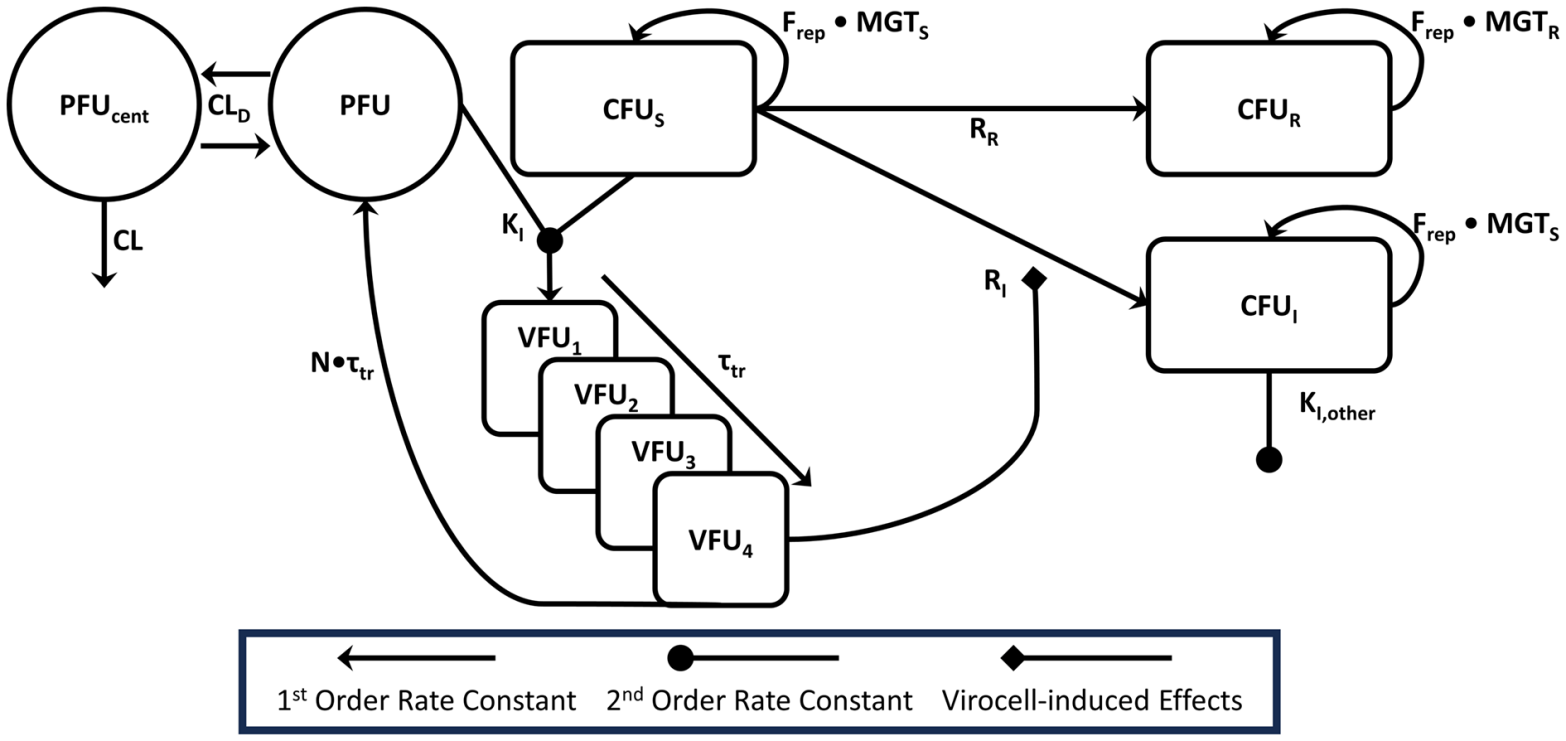
Model diagram this figure is representative for a single phage that infects a given bacterial sub-population. Our platform mechanism-based mathematical model of phage action accounts for the observed phage pharmacokinetics, including the distributional clearance (CL_D_) between the central reservoir and extracapillary space. Phage (PFU) and bacteria (CFU_S_ or other sensitive sub-populations) were expected to intermingle in the extracapillary space and bind with rate constant *K*_I_, indicated by the merging lines connecting the PFU and CFU_S_ compartments. Virocells mature through 4 transit compartments with a total transit time of *τ*_tr_. Virocells in the final compartment (VFU_4_) were modeled as inducing the adaptive resistance process, which would convert pan-sensitive cells into either single-phage resistant mutants (CFU_I_) or double phage resistant mutants (CFU_R_). For combination therapy, single-phage resistant PAO1 mutants could be acted on by the other phage in an identical manner. Phage–phage interactions were tested empirically on the adsorption rate constant and included based on likelihood ratio testing. For model parsimony, phage–phage interactions were driven by the same virocell-induced effect process that governs adaptive resistance. Bacteria were all modeled as growing logistically (F_rep_) with a mean generation time (MGT_S_) for phage-sensitive or single-resistant phages subpopulations or MGTR for 3-phage resistant subpopulations.

**Figure 3:**
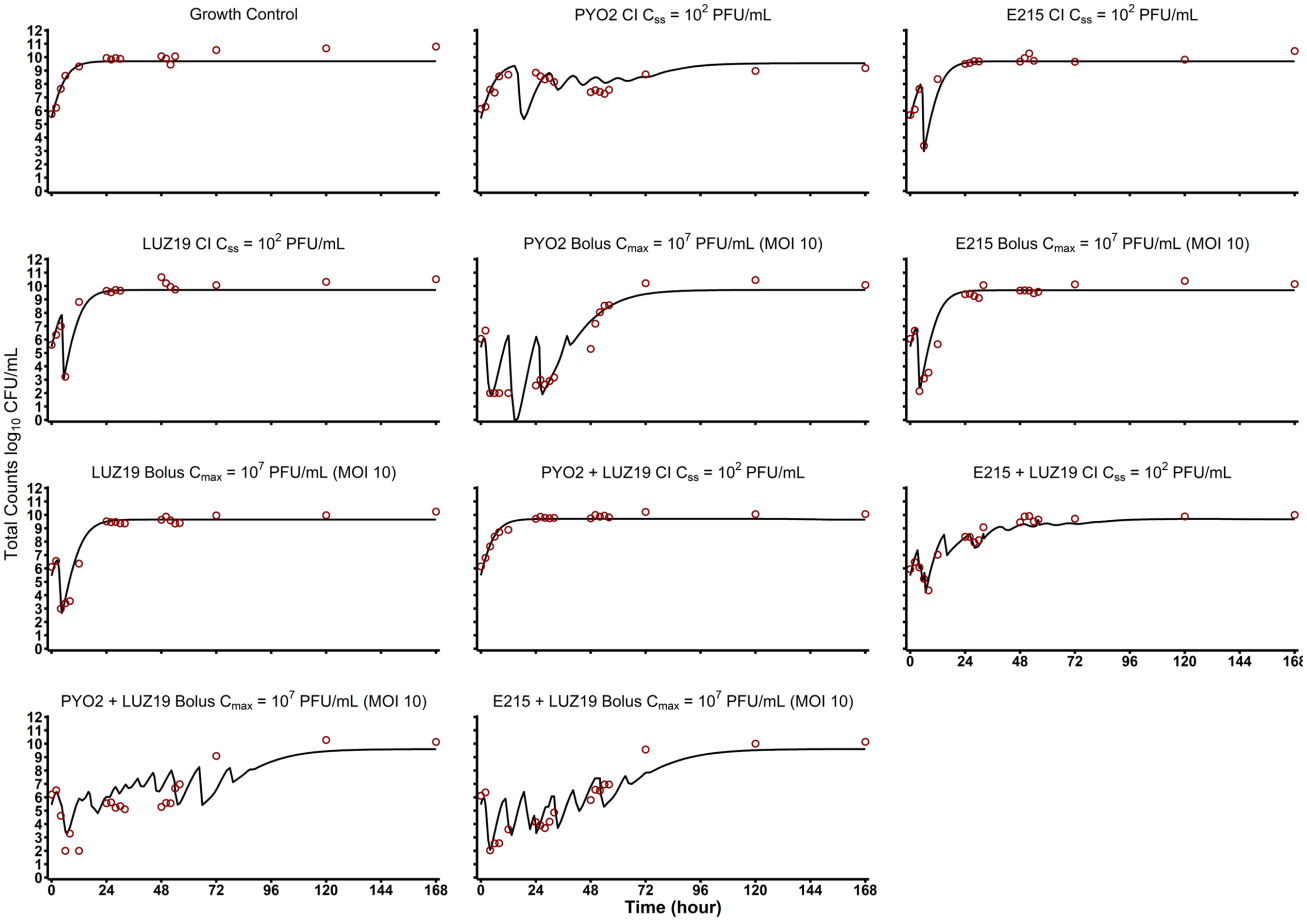
*Post Hoc* model fits and observed versus predicted plots. Here the individual model fits are depicted for each of the HFIM experimental arms. Combination therapies are graphed as averaged values of the log-transformed concentrations (*n* = 2). Quantification limits were plotted at 10^2^ CFU/mL and treated as censored data in the mechanism-based mathematical modeling.

The data were well described by the modified SIR model (see Figure 2) that incorporated susceptible, infected, and adaptively resistant cell subpopulations as indicated by objective model fitting criteria (Table 2; Supplementary Figure S2). *Post Hoc* fits (Figure 3) show bacterial counts for all regimens to be well characterized. For both all phage susceptible and single-phage resistant subpopulations, the MGT of cells were estimated at 23.1 min (3.73% RSE) and MGT of double phage-resistant subpopulation were estimated at 56.6 min (4.36% RSE) to account for growth fitness loss.

**Table 2:** Parameter estimates for mechanism-based PD model.

Parameter	Definition	Units	Estimate (%RSE^a^)
**Pharmacokinetic parameters**			
CL_D,PYO_	Distributional clearance of PYO2^b^	mL/h	2.96 (20.2%)
CL_D,215_	Distributional clearance of E215^b^	mL/h	3.66 (19.4%)
CL_D,LUZ_	Distributional clearance of LUZ19^b^	mL/h	2.42 (13.9%)
**Bacterial-specific parameters**			
LGCFUMX	System maximum capacity^b^	log_10_ (CFU/mL)	9.69 (0.603%)
*γ*	Shape parameter for logistic growth^c^	–	0.1 (FIXED)
LGINOC	Starting inoculum^b^	log_10_ (CFU/mL)	5.46 (1.64%)
MGT_S_	Mean generation time for susceptible- or single phage-resistant subpopulation^b^	min	23.1 (3.73%)
MGT_R_	Mean generation time for double phage-resistant subpopulation^b^	min	56.6 (4.36%)
**Shared phage parameters**			
LGR_r_	Log-transformed 1st order rate constant for adaptive resistance to all three phages^c^	log_10_ (1/h)	−8.60 (0.694%)
LGEC_50_	Log-transformed concentration for virocells-induced resistance/collateral phage effects^c^	log_10_ (CFU/mL)	2 (FIXED)
h_rst_	Shape parameter for virocells-induced effects^c^	–	5 (FIXED)
LGK_rev_	Log-transformed 1st order rate constant for reversion of resistant cells to susceptible^c^	log_10_ (1/h)	−2.75 (3.52%)
**PYO2-specific parameters**			
LGK_pyo_	Log-transformed 2nd order rate constant for phage-bacteria binding^c^	log_10_ (mL/PFU/h)	−9.64 (0.582%)
LGR_pyo_	Log-transformed 1st order rate constant for single-phage adaptive resistance^c^	log_10_ (1/h)	−15 (FIXED)
*τ*_pyo_	Mean transit time for virocells maturation^b^	min	20.4 (21.2%)
*N*_pyo_	PYO2 burst size^b^	PFU/CFU	73.8 (25.1%)
E215-specific parameters			
LGK_215_	Log-transformed 2nd order rate constant for phage-bacteria binding^c^	log_10_ (mL/PFU/h)	−9.48 (0.0968%)
LGR _215_	Log-transformed 1st order rate constant for single-phage adaptive resistance^c^	log_10_ (1/h)	−6.17 (7.56%)
*τ*_215_	Mean transit time for virocells maturation^b^	min	31.7 (13.6%)
*N*_215_	PYO2 burst size^b^	PFU/CFU	168 (12.5%)
E_max,EoL_	Maximum effect of E215 on LUZ19 adsorption rate^c^	–	4.46 (5.82%)
**LUZ19-specific parameters**			
LGK_luz_	Log-transformed 2nd order rate constant for phage-bacteria binding^c^	log_10_ (mL/PFU/h)	−8.43 (0.0973%)
LGR_luz_	Log-transformed 1st order rate constant for single-phage adaptive resistance^c^	log_10_ (1/h)	−5.68 (4.68%)
*τ*_luz_	Mean transit time for virocells maturation^b^	min	27.9 (8.69%)
*N*_luz_	PYO2 burst size^b^	PFU/CFU	83.0 (18.0%)
E_max,LoE_	Maximum effect of LUZ19 on E215 adsorption rate^c^	–	8.48 (13.3%)
E_max,LoP_	Maximum effect of LUZ19 on PYO2 adsorption rate^c^	–	1.41 (15.9%)
**Residual variability**			
a_PAO1_	Constant residual variability for PAO1	log_10_ (CFU/mL)	0.83 (4.01%)
a_pyo2_	Constant residual variability for PYO2 in the HFIM cartridge	log_10_ (PFU/mL)	1.85 (7.90%)
a_pyo2,cent_	Constant residual variability for PYO2 in the central reservoir	log_10_ (PFU/mL)	3.30 (6.94%)
a_E215_	Constant residual variability for E215 in the HFIM cartridge	log_10_ (PFU/mL)	2.34 (6.57%)
a_E215,cent_	Constant residual variability for E215in the central reservoir	log_10_ (PFU/mL)	2.33 (6.80%)
a_LUZ19_	Constant residual variability for LUZ19 in the HFIM cartridge	log_10_ (PFU/mL)	1.67 (5.61%)
a_LUZ19,cent_	Constant residual variability for LUZ19 in the central reservoir	log_10_ (PFU/mL)	2.17 (5.39%)

a RSE% – residual standard error percent.

b IIV fixed to *ω* = 0.05.

c IIV fixed to *ω* = 0.

Cell adsorption rates for PYO2, E215, and LUZ19 were estimated using the SIR model as −9.64 (0.582% RSE), −9.48 (0.0968% RSE), and −8.43 (0.0973% RSE) log_10_ (mL•PFU^−1^•h^−1^), respectively. E215 was estimated as having a burst size of 168 PFU/CFU, which was significantly larger than the estimated PYO2 burst size of 73.8 PFU/CFU or estimated LUZ19 burst size of 83.0 PFU/CFU. These extrapolated phage properties were in accordance with previously values for these phages (Ceyssens et al., 2011; Forti et al., 2018).

### Monte Carlo simulation of phage dosing strategies

**Figure 4:**
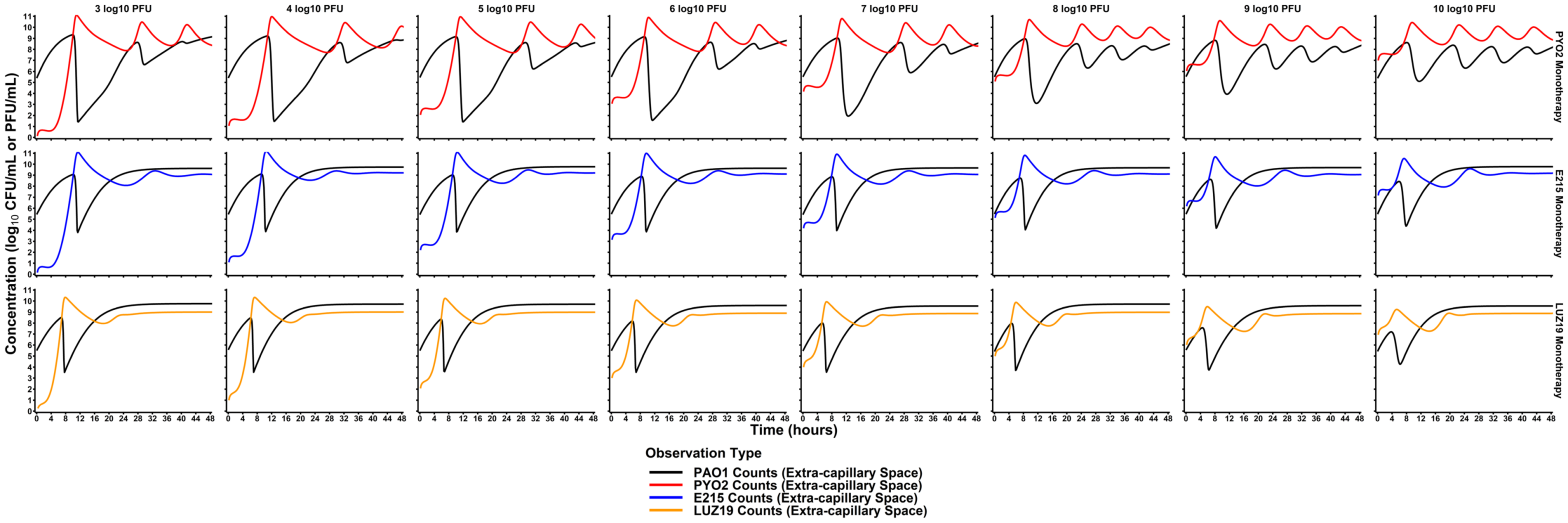
Comparison of dose effects on PAO killing. Monte Carlo simulations were performed at each of the listed doses (columns) for each phage monotherapy (rows) based on the median PD profiles predicted by the mechanism-based model. Simulations of monotherapy regimens predicted that phages could provide therapeutic benefit even at doses significantly lower than the bacterial burden. This is indicated by the significant decrease in concentration even with low doses (3–5 log_10_ PFU). Compared to dose-fractionation simulations (Supplemental material), initial dose level was more influential on bacterial killing. Paradoxically, the extent of bacterial killing is predicted to decrease as PYO2 dose increases with more frequent predator–prey cycling. Ultimately, these simulation-based studies are critical for translating phages for human use and designing future studies efficiently.

Simulations of dose escalation strategies from 3 to 10 log_10_ (PFU) per day showed varying effects that were phage strain-dependent (Figure 4). PYO2 treatment was predicted to produce a more significant reduction in bacterial counts, but after a longer latent period of activity. By comparison, both E215 and LUZ19 showed similar extents to bacterial killing despite increasing the phage dose by 7 orders of magnitude. Outside to dose effects on extent of bacterial killing, dose also influenced the rate of predator–prey cycling observed for PYO2, with larger doses predicted to produce an increased frequency of the bacterial killing-regrowth cycle. Utilizing typical dose fractionation studies, dividing a daily dose of 7 log_10_ (PFU)/d across multiple individual doses resulted in a negligible impact on the extent of bacterial killing or the rate of predator–prey cycling (Supplementary Figure S3). Concentration of phages in simulations of dose fractionation were consistently higher than the expected *C*_max_ from administration alone, indicating that the endogenous phage production is expected to obfuscate the exogenous administration for all dose fractionation strategies.

## Discussion

This study is the first to quantify the full time-course of phage PD in the HFIM, which was then used to identify optimal target concentrations for a three-phage cocktail using a hybrid ML-PK/PD approach. Current best practice recommendations acknowledge many gaps in the foundational knowledge of phage PK/PD for clinical use in humans (Suh et al., 2022). This study investigated three clinically relevant *Pseudomonas* virulent phages *in vitro* under dynamic conditions to quantitatively assess the PD of mono- and 2-phage cocktail treatments. We used the HFIM to explore expected phage TMPD over clinically relevant time periods. Altogether, the most relevant aspects of TMPD relate to self-dosing at the site of infection, second-order phage-bacteria binding processes, maturation of virocells, and phage–phage interactions, which can be quantified *in vitro* for pre-clinical assessment. Through mathematical modeling, we found that adsorption rate, latent period, and burst size were significant factors affecting TMPD of an individual phage, which themselves are dependent on complex biochemical processes. Further refinement of model-based approaches to quantify and assess TMPD will be critical to the establishment, testing, and implementation of N-phage cocktails.

The decision to utilize bolus or continuous infusions was principally driven by the need to utilize dosage regimens that could discriminate between exogenous administration and endogenous production of phages. Specifically, *absent* endogenous production of phage, bolus dosing is expected to produce a single maximum concentration, whereas continuous infusion regimens are expected to produce a steady-state concentration after about 10 h. However, both bolus and continuous infusion regimens will eventually lead to phage concentrations that are entirely driven by endogenous production on susceptible cells *in situ*. To that end, the HFIM successfully was able to replicate target pharmacokinetic profiles based on previous *in vivo* studies, as confirmed by PK profile of phage bolus doses within the first hours of treatment. For 2-phage cocktails (e.g., E215 + LUZ19 and PYO2 + LUZ19), duplicate continuous infusion experiments showed reduced bacterial killing as compared to the bolus regimens. Though the true mechanism of antagonism is likely to be complex, assessing this interaction as a single parameter on adsorption creates a foundational model structure that more readily compares different phage strains. Previous studies have shown that bacteria can undergo expressional changes to adaptively resist phage action, which was found to improve description of predator–prey cycling throughout (Jacobs et al., 2016; Jurado et al., 2022).

*In vitro* PYO2, E215, and LUZ19 parameters of infectivity including burst sizes and latent periods agreed with the model estimates trained using bacteria and phage density patterns in the HFIM (Ceyssens et al., 2011; Forti et al., 2018). Given expected differences in growth conditions between the HFIM and the *in vitro* methods to quantify phage parameters of infectivity (e.g., burst size, latent period, adsorption rate, etc.), being able to accurately assess burst size and latent time with HFIM data may facilitate development of future phage dosing strategies and target concentrations. Modeling approaches with delayed differential equations (DDE) are an alternative analytical strategy to describe phage activity (Cairns et al., 2009). Initial versions of predator–prey models were explored using the DDE solver within Monolix. However, model run times were significantly longer with increased rates of premature termination (data not shown). Therefore, instability and computational cost made an ODE-based model preferable for this study. Furthermore, underlying stochasticity in the timing and extent of killing and re-growth make optimizing phage therapy challenging. Using nonlinear mixed effects modeling can assess and quantify inter-experimental variability through estimation of random effects on select phage parameters, which can then be used hypothesize future studies through Monte Carlo simulations.

Simulations utilizing the mechanism-based model showed significant influence of daily dose on antibacterial PD. Dose effects were phage strain-specific, with PYO2 paradoxically reducing bacterial counts more significantly with lower daily doses. Given the phage strain-specific nature to dosing, this may indicate that cocktail-based strategies may require optimization of the dose for each phage in the cocktail rather than utilizing a uniform dose. Separately, dose fractionation of identical daily doses across multiple daily dosing showed negligible influence on the extent of bacterial killing. Altogether, these results would support dosing strategies that obtain a phage-specific *C*_max_ after the first dose for an N-phage cocktail, with subsequent doses having a less impactful role in overall treatment efficacy. In the context of TMPD, dosing strategies that achieve a target *C*_max_ after the first dose will be predominately dictated by phage PK from exogenous administration rather than endogenous production, initially.

This study is principally limited by not developing models based on MDR *P. aeruginosa* isolates, which would be more clinically relevant to the types of isolates phage therapy is typically employed. However, given limited availability of data on the correlation between resistance to small molecule antibiotics and phage infectivity, it is unclear whether MDR-status impacts N-phage cocktail efficacy. Given that most phage action on clinical isolates is typically quantified relative to the phages’ isolation host for efficiency of plating studies, future studies could seek to incorporate these metrics as PD covariates to improve model extrapolation (Smith et al., 2021). Variability in phage-bacterial interactions will influence the performance of a given regimen. Future studies should include assessment of multiple, clinical bacterial isolates to better characterize random effects on phage-specific parameters of infectivity. This will allow better assessment of regimens that incorporate observed variability. Characterization of human PK will ultimately be required to identify dosage regimens. The mathematical model of phage action developed in this study addressed bacterial resistance as being driven by number of virocells; this was done for model parsimony as it minimized the number of parameters needed to address resistance development. Bacterial resistance to phage is largely driven by pre-existing mutations which are selected for after phage exposure, though adaptive resistance is still a relevant concern. Nonetheless, these preliminary data aid in developing a strategy to identify target concentrations. Advancing *in vitro*, animal-sparing studies, to characterize the PK/PD properties of phages will be critically important to maximizing the therapeutic potential of phages as a class of antimicrobials.

## Data availability statement

The raw data supporting the conclusions of this article will be made available by the authors, without undue reservation and after execution of a Data Use Agreement.

## Author contributions

NS: Conceptualization, Data curation, Formal analysis, Funding acquisition, Investigation, Methodology, Project administration, Resources, Software, Supervision, Validation, Visualization, Writing – original draft, Writing – review & editing. TN: Conceptualization, Data curation, Investigation, Methodology, Writing – review & editing. WC: Conceptualization, Data curation, Investigation, Methodology, Writing – review & editing. JS: Conceptualization, Data curation, Investigation, Methodology, Writing – review & editing. HS: Data curation, Investigation, Methodology, Writing – review & editing. BH: Data curation, Investigation, Methodology, Writing – review & editing. TL: Conceptualization, Investigation, Methodology, Writing – review & editing. DR: Conceptualization, Data curation, Investigation, Methodology, Writing – original draft, Writing – review & editing.
