# Supplementary material for: A mechanism-based pathway toward administering highly active N-phage cocktails
Source: Front Microbiol. 2023 Nov 15;14:1292618. doi: 10.3389/fmicb.2023.1292618 (PMC10690594; doi:10.3389/fmicb.2023.1292618)

SUPPLEMENTAL MATERIALS

A Mechanism-based Pathway Toward Administering Highly Active N-Phage Cocktails

Nicholas M. Smith[1,#], Thoms D. Nguyen[1], Wai Hoe Chin[2], Jacob Sanborn[1], Harriet de Souza[1],Brian M. Ho[1], Tiffany Luong[2], Dwayne R. Roach[2]

[1]Division of Clinical and Translational Therapeutics, School of Pharmacy & Pharmaceutical Sciences, University at Buffalo, Buffalo, New York, USA; [2]Department of Biology, San Diego State University, San Diego , California, USA

[#]Correpsondence:

Nicholas M. Smith, PharmD, PhD

308 Farber Hall

Buffalo, New York, 14203

nmsmith2@buffalo.edu

Figure S1 Hollow Fiber Infection Model Results.

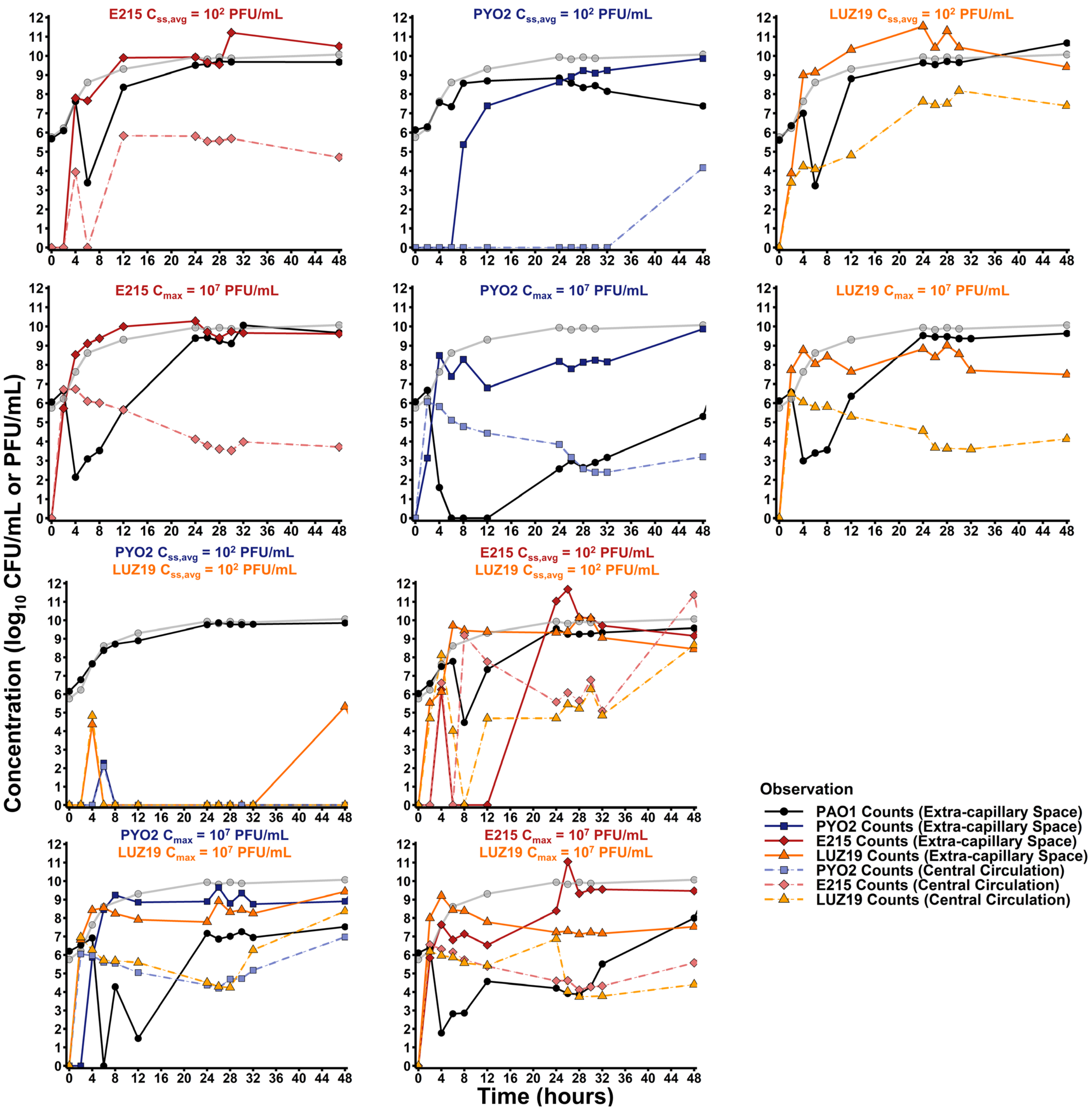

Equations:

Parameter Transformations	
$$k_{tr,pyo} = \frac{4}{\frac{\tau_{pyo}}{60}}$$	**1**
$$k_{tr,215} = \frac{4}{\frac{\tau_{215}}{60}}$$	**2**
$$k_{tr,luz} = \frac{4}{\frac{\tau_{luz}}{60}}$$	**3**
$$k_{gs} = \frac{60}{MGT_S}$$	**4**
$$k_{gr} = \frac{60}{MGT_R}$$	**5**
$$EC_{50} = 10^{LGEC_{50}}$$	**6**
$$k_{i,pyo} = 10^{LGK_{\mathrm{i,pyo}}}$$	**7**
$$k_{i,215} = 10^{LGK_{\mathrm{i,215}}}$$	**8**
$$k_{i,luz} = 10^{LGK_{\mathrm{i,luz}}}$$	**9**
$$r_{pyo} = 10^{LGR_{pyo}}$$	**10**
$$r_{215} = 10^{LGR_{215}}$$	**11**
$$r_{luz} = 10^{LGR_{luz}}$$	**12**
$$r_r = 10^{LGR_r}$$	**13**
$$K_{rev} = 10^{LGKrev}$$	**14**
$$IC_s = 10^{INOC}$$	**15**
Secondary Parameter Equations	
$$PFU_{\mathrm{cent,pyo}} = \frac{APFU_{\mathrm{cent,pyo}}(t)}{V_{cent}}$$	**17**
$$PFU_{\mathrm{cent,215}} = \frac{APFU_{\mathrm{cent,215}}(t)}{V_{cent}}$$	**18**
$$PFU_{\mathrm{cent,luz}} = \frac{APFU_{\mathrm{cent,luz}}(t)}{V_{cent}}$$	**19**

$$PFU_{pyo} = \frac{APFU_{pyo}(t)}{V_{extra}}$$	**20**
$$PFU_{215} = \frac{APFU_{215}(t)}{V_{extra}}$$	**21**
$$PFU_{\mathrm{luz}} = \frac{APFU_{\mathrm{luz}}(t)}{V_{extra}}$$	**22**
$$CFU_{tot} = CFU_L(t) + CFU_S(t) + CFU_{I1}(t) + CFU_{I2}(t) + CFU_R(t)$$	**23**
$$F_{rep} = \left(1 - \left(\frac{CFU_{tot}}{CFU_{\max}}\right)^{\gamma}\right)$$	**24**
$$E_{pyo} = \frac{VFU_{pyo,4}^{h}(t)}{EC_{50,\mathrm{rst,pyo}}^{h} + VFU_{pyo,4}^{h}(t)}$$	**25**
$$E_{215} = \frac{VFU_{215,4}^{h}(t)}{EC_{50,\mathrm{rst},215}^{h} + VFU_{215,4}^{h}(t)}$$	**26**
$$E_{luz} = \frac{VFU_{luz,4}^{h}(t)}{EC_{50,\mathrm{rst},luz}^{h} + VFU_{luz,4}^{h}(t)}$$	**27**
$$K_{pyo} = k_{\mathrm{i,pyo}} \cdot PFU_{pyo} \cdot (1 + E_{\mathrm{max,LoP}} \cdot E_{luz})$$	**28**
$$K_{215} = k_{\mathrm{i},215} \cdot PFU_{215} \cdot (1 + E_{\mathrm{max,LoE}} \cdot E_{luz})$$	**29**
$$K_{luz} = k_{\mathrm{i},luz} \cdot PFU_{luz} \cdot (1 + E_{\mathrm{max,EoL}} \cdot E_{215})$$	**30**
$$R_{\mathrm{pyo}} = r_{pyo} \cdot E_{pyo}$$	**31**
$$R_{215} = r_{215} \cdot E_{215}$$	**32**
$$R_{\mathrm{luz}} = r_{\mathrm{luz}} \cdot E_{\mathrm{luz}}$$	**33**
$$R_{r,\mathrm{pyo}} = r_r \cdot E_{pyo}$$	**34**
$$R_{r,215} = r_r \cdot E_{215}$$	**35**
$$R_{r,\mathrm{luz}} = r_r \cdot E_{\mathrm{luz}}$$	**36**
ODE for Bacteria	
$$\frac{dCFU_S}{dt} = F_{rep} \cdot K_{gs} \cdot CFU_S - \left(K_{pyo} + K_{215} + K_{luz} + R_{\mathrm{pyo}} + R_{215} + R_{\mathrm{luz}} + R_{\mathrm{r,pyo}} + R_{\mathrm{r},215} + R_{\mathrm{r,luz}}\right) \cdot CFU_S$$	**37**
$$CFU_s(t = 0) = 0$$	**38**
$$\frac{dCFU_{I1}}{dt} = F_{rep} \cdot K_{gs} \cdot CFU_{I1} - (K_{pyo} + K_{215}) \cdot CFU_{I1} + R_{luz} \cdot CFU_S$$	**39**

$$\frac{dCFU_{I1}}{dt} = 0$$	**40**
$$\frac{dCFU_{I2}}{dt} = F_{rep} \cdot K_{gs} \cdot CFU_{I2} - K_{luz} \cdot CFU_{I2} + (R_{\mathrm{pyo}} + R_{215}) \cdot CFU_S$$	**41**
$$CUF_{I2}(t = 0) = 0$$	**42**
$$\frac{dCFU_R}{dt} = F_{rep} \cdot K_{gr} \cdot CFU_R + \left(R_{\mathrm{r,pyo}} + R_{\mathrm{r,215}} + R_{\mathrm{r,luz}}\right) \cdot CFU_S$$	**43**
$$CFU_R(t = 0) = 0$$	**44**
ODEs for PYO2	
$$\frac{dAPFU_{cent,pyo}}{dt} = IN_{pyo}(t) + Q_{pyo} \cdot \left(PFU_{\mathrm{pyo}} - PFU_{pyo,\mathrm{cent}}\right) - CL \cdot PFU_{\mathrm{pyo,cent}}$$	**45**
$$\frac{dAPFU_{pyo}}{dt} = N_{pyo} \cdot K_{tr,pyo} \cdot VFU_{pyo,4} \cdot V_{extra} - K_{pyo} \cdot PFU_{pyo} \cdot (CFU_S + CFU_{I1}) + Q_{pyo} \cdot \left(PFU_{\mathrm{pyo,cent}} - PFU_{pyo}\right)$$	**46**
$$\frac{dVFU_{\mathrm{pyo,1}}}{dt} \cdot PFU_{pyo} \cdot (CFU_S + CFU_{I1}) - K_{\mathrm{tr,pyo}} \cdot VFU_{\mathrm{pyo,1}}$$	**47**
$$\frac{dVFU_{\mathrm{pyo,2}}}{dt} = K_{\mathrm{tr,pyo}} \cdot (VFU_{\mathrm{pyo,1}} - VFU_{\mathrm{pyo,2}})$$	**48**
$$\frac{dVFU_{\mathrm{pyo,3}}}{dt} = K_{\mathrm{tr,pyo}} \cdot (VFU_{\mathrm{pyo,2}} - VFU_{\mathrm{pyo,3}})$$	**49**
$$\frac{dVFU_{\mathrm{pyo,4}}}{dt} = K_{\mathrm{tr,pyo}} \cdot (VFU_{\mathrm{pyo,3}} - VFU_{\mathrm{pyo,4}})$$	**50**
ODEs for E215	
$$\frac{dAPFU_{\mathrm{cent,215}}}{dt} = IN_{215}(t) + Q_{215} \cdot \left(PFU_{215} - PFU_{\mathrm{215,cent}}\right) - CL \cdot PFU_{\mathrm{215,cent}}$$	**51**
$$\frac{dAPFU_{215}}{dt} = N_{215} \cdot K_{tr,215} \cdot VFU_{215,4} \cdot V_{extra} - K_{215} \cdot PFU_{215} \cdot (CFU_S + CFU_{I1}) + Q_{215} \cdot \left(PFU_{\mathrm{215,cent}} - PFU_{215}\right)$$	**52**
$$\frac{dVFU_{215,1}}{dt} = K_{215} \cdot PFU_{215} \cdot (CFU_S + CFU_{I1}) - K_{\mathrm{tr,215}} \cdot VFU_{215,1}$$	**53**
$$\frac{dVFU_{215,2}}{dt} = K_{\mathrm{tr,215}} \cdot (VFU_{215,1} - VFU_{215,2})$$	**54**
$$\frac{dVFU_{215,3}}{dt} = K_{\mathrm{tr,215}} \cdot (VFU_{215,2} - VFU_{215,3})$$	**55**
$$\frac{dVFU_{215,4}}{dt} = K_{\mathrm{tr,215}} \cdot (VFU_{215,3} - VFU_{215,4})$$	**56**

ODEs for LUZ19	
$$\frac{dAPFU_{\text{cent.luz}}}{dt} = IN_{\text{luz}}(t) + Q_{\text{luz}} \cdot \left(PFU_{\text{luz}} - PFU_{\text{luz,cent}}\right) - CL \cdot PFU_{\text{luz,cent}}$$	**57**
$$\frac{dAPFU_{\text{luz}}}{dt} = N_{\text{luz}} \cdot K_{tr,\text{luz}} \cdot VFU_{\text{luz},4} \cdot V_{extra} - K_{\text{luz}} \cdot PFU_{\text{luz}} \cdot (CFU_S + CFU_{I1}) + Q_{\text{luz}} \cdot \left(PFU_{\text{luz,cent}} - PFU_{\text{luz}}\right)$$	**58**
$$\frac{dVFU_{\text{luz},1}}{dt} = K_{\text{luz}} \cdot PFU_{\text{luz}} \cdot (CFU_S + CFU_{I1}) - K_{\text{tr,luz}} \cdot VFU_{\text{luz},1}$$	**59**
$$\frac{dVFU_{\text{luz},2}}{dt} = K_{\text{tr,luz}} \cdot (VFU_{\text{luz},1} - VFU_{\text{luz},2})$$	**60**
$$\frac{dVFU_{\text{luz},3}}{dt} = K_{\text{tr},215} \cdot (VFU_{\text{luz},2} - VFU_{\text{luz},3})$$	**61**
$$\frac{dVFU_{\text{luz},4}}{dt} = K_{\text{tr,luz}} \cdot (VFU_{\text{luz},3} - VFU_{\text{luz},4})$$	**62**

Figure S2: Observed versus individual predicted plots. Each panel represents the observed versus predicted plot for each observation type including concentrations of PAO1, PYO2, E215, and LUZ19 in the HFIM cartridge and PYO2, E215, and LUZ19 concentrations in the central reservoir. Overall, the model performed well, but had a more challenging time fitting data in the central reservoir once phage was being endogenously produced within the extracapillary space. Axes are plotted in terms of log_{10}(PFU/mL) or log_{10}(CFU/mL) as appropriate.

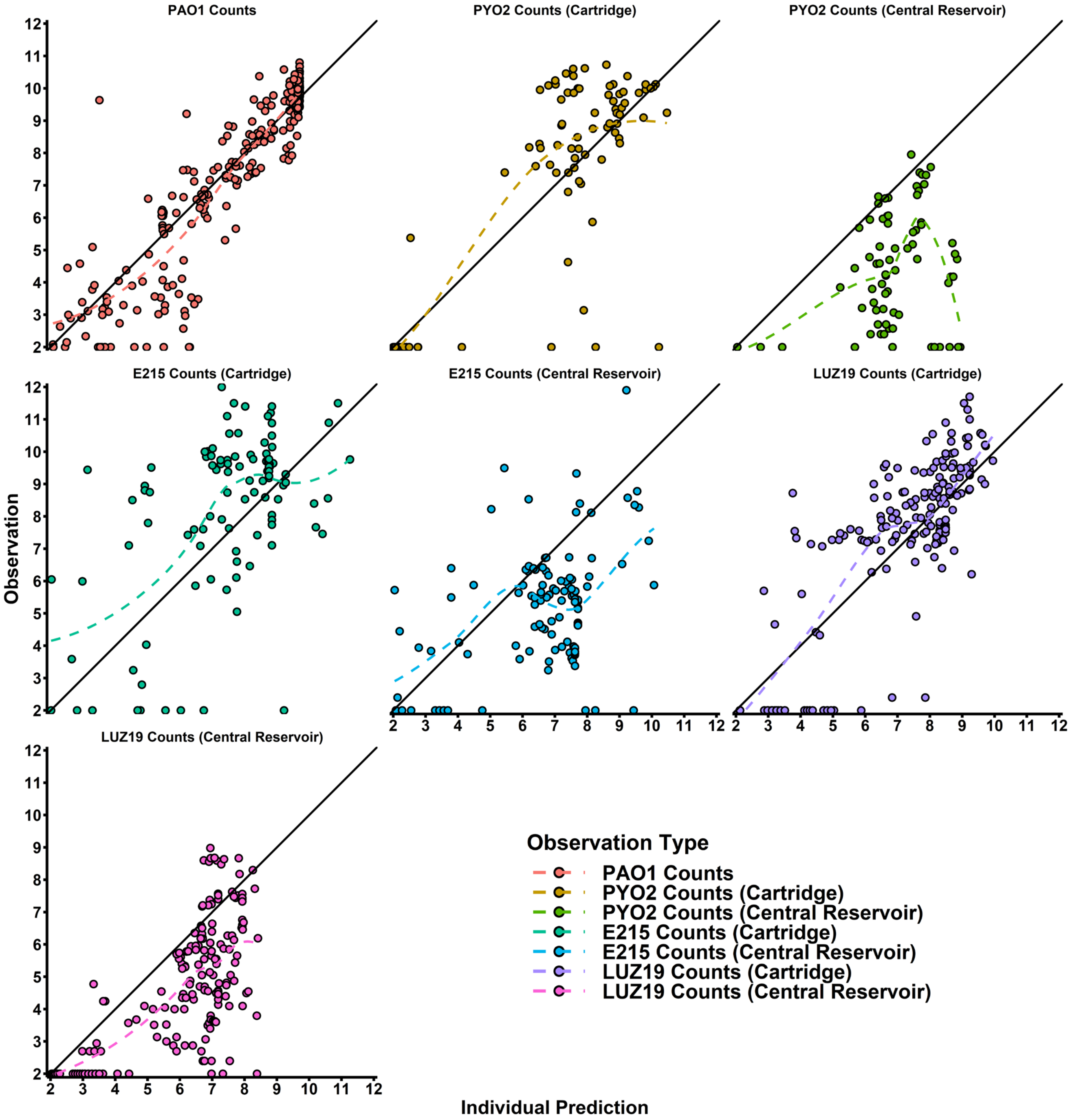

Figure S3: Comparison of Dose Fractionation on PA01 Killing. Monte Carlo simulations of monotherapy regimens utilizing identical 7 $\log_{10}$ PFU doses, but divided into Q24H, Q12H, Q8H, Q6H, or Q4H dosing. Across all three phages, dose fractionated produced negligible effects on bacterial killing, providing evidence of limited utility in using multiple dosing strategies. Individual dosing events were obscured by the high endogenous production of phages throughout the simulation, reinforcing the concept that the initial dose may be the most critical factor in reducing bacterial burden.

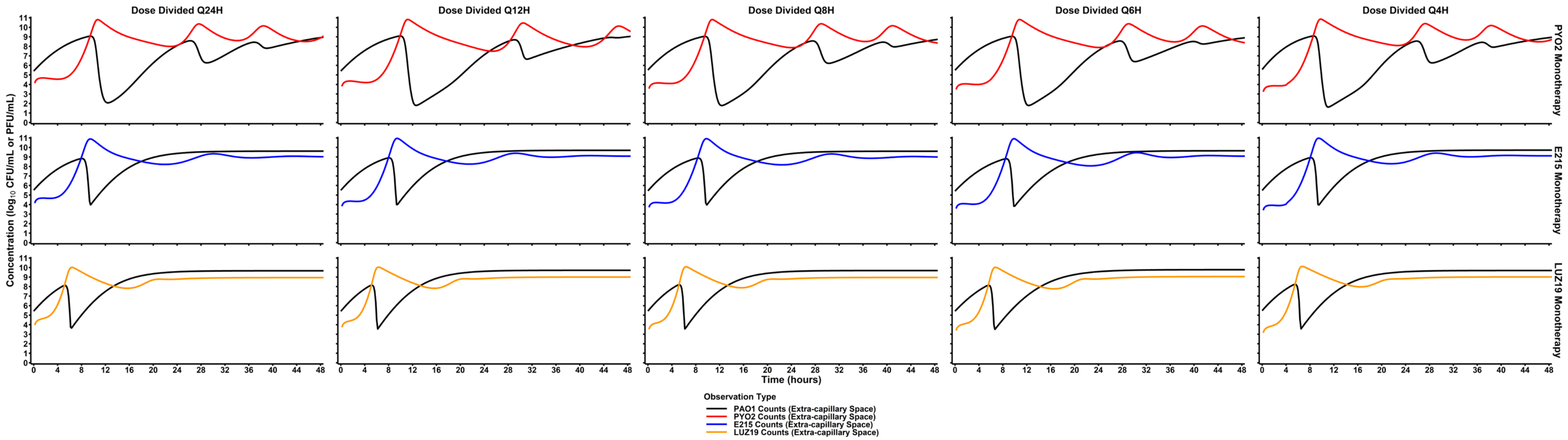

Supplement: Supplementary file 1 [file Data_Sheet_1.pdf]
